# The complete mitochondrial genome of *Crossocheilus siamensis* (Cyprinoidea: cyprinidae)

**DOI:** 10.1080/23802359.2016.1225530

**Published:** 2016-09-12

**Authors:** Huizhi Sun, Tianqing Huang, Xingran Wang, Yanna Wang, Ying Han

**Affiliations:** Department of Animal Science and Technology, Northeast Agricultural University, Harbin, Heilongjiang, China

**Keywords:** *Crossocheilus siamensis*, mitochondrial genome, phylogeny

## Abstract

In this study, the complete mitochondrial genome of *Crossocheilus siamensis* has been determined by polymerase chain reaction methods for the first time. The overall base composition of *C. siamensis* mitogenome is 30.4% for A, 25.5% for C, 17.1% for G, and 27.0% for T. The percentage of G + C content is 42.6%. The mitogenome is a circular DNA molecule of 16,611bp in length with a D-loop region and contains 22 transfer RNA (tRNA) genes, 2 ribosomal RNA (rRNA) genes, and 13 protein-coding genes. The mitochondrial genome sequencing for *C. siamensis* in this study provides important molecular data for further evolutionary analysis for Cyprinoidea.

*Crossocheilus siamensis*, which belongs to order Cyprinoidea, family Cyprinidae, genus Crossocheilus is a species of freshwater fish with slender body, beige, and with a special black horizontal stripe. It is mainly found in the Great Mekong area of Southeast Asian countries.

In this study, *C. siamensis* samples were collected from Xayaburi City of Laos (19°15′N; 101°48′E). The specimen is stored in Northeast Agricultural University and its accession number is PS71007003000400. The genome DNA was extracted following the traditional phenol–chloroform method (Taggart et al. 1992). Twenty-six primers were designed to amplify the PCR products for sequencing. The sequencing results were then assembled using ContigExpress 9.0 software (New York, NY). The transfer RNA (tRNA) genes were identified using the program tRNAscan-SE 1.21 (http://lowelab.ucsc.edu/tRNAscan-SE). The locations of protein-coding genes were determined by comparing with the corresponding known sequences of other *Crossocheilus* fish species.

The complete mitochondrial genome length of *C. siamensis* was 16,611bp in length (GenBank accession number KX686103). It consisted of 13 protein-coding genes, 2 rRNA genes, 22 tRNA genes, and 1 D-loop region. The overall base composition of the mitogenome is 30.4% for A, 25.5% for C, 17.1% for G, and 27.0% for T. The percentage of G + C content is 42.6%. To validate the phylogenetic position of *C. siamensis*, we perform multiple sequence alignment and MEGA 6.0 (Tamura et al. 2013) to construct a maximum-likelihood (ML) tree containing complete mitochondrial genome DNA of 14 species in Cyprinoidea. As shown in the phylogenetic tree (Figure 1), our sequence was clustered in the genus *Crossocheilus*, including *C. atrilimes* and *C. langei*.

**Figure 1.**
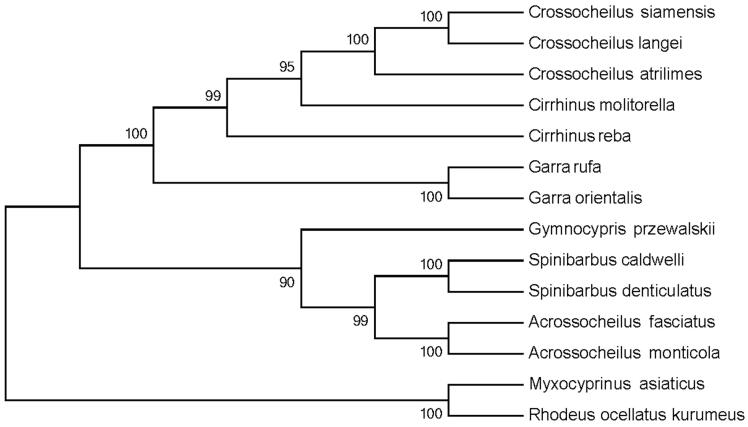
An ML tree of the 14 species from Cyprinoidea was constructed based on complete mitochondrial genome data. The analyzed species and corresponding NCBI accession numbers are as follows: *C. siamensis* (KX686103), *C. langei* (NC_029443.1), *C. atrilimes* (NC_029447.1), *Cirrhinus molitorella* (AP011390.1), *C. reba* (NC_029445.1), *Garra rufa* (NC_022941.1), *G. orientalis* (AP011202.1), *Gymnocypris przewalskii* (NC_019604.1), *Spinibarbus caldwelli* (NC_022149.1), *S. denticulatus* (AP013335.1), *Acrossocheilus fasciatus* (KF781289.1), *A.s monticola* (NC_022145.1), *Myxocyprinus asiaticus* (AY526869.1), *Rhodeus ocellatus kurumeus* (AB070205.1).
